# {*N*,*N*′-Bis[1-(pyridin-2-yl)ethyl­idene]­propane-1,3-diamine}­bromidocopper(II) tetra­fluoridoborate

**DOI:** 10.1107/S1600536811022513

**Published:** 2011-06-11

**Authors:** Li-Jun Liu

**Affiliations:** a Experimental Center, Linyi University, Linyi Shandong 276005, People’s Republic of China

## Abstract

In the title compound, [CuBr(C_17_H_20_N_4_)]BF_4_, the Cu^II^ ion is five-coordinated by the four N atoms of the tetra­dentate Schiff base ligand and by one bromide ion, thereby forming a square-pyramidal CuN_4_Br coordination geometry. The dihedral angle between the pyridine rings of the Schiff base is 54.39 (18)°. In the crystal, the components are linked by C—H⋯F inter­actions.

## Related literature

For a related structure and background references, see: Liu (2011 ▶).
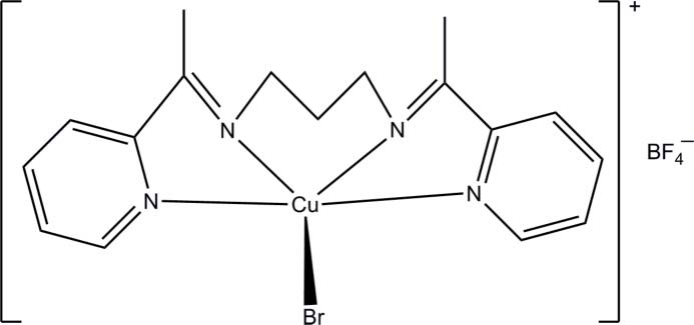


## Experimental

### 

#### Crystal data

[CuBr(C_17_H_20_N_4_)]BF_4_
*M* *_r_* = 510.63
Triclinic, 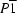

*a* = 8.2508 (17) Å
*b* = 8.9511 (18) Å
*c* = 13.158 (3) Å
α = 92.391 (2)°
β = 94.847 (2)°
γ = 96.422 (2)°
*V* = 960.9 (3) Å^3^
*Z* = 2
Mo *K*α radiation
μ = 3.26 mm^−1^
*T* = 298 K
0.27 × 0.27 × 0.23 mm

#### Data collection

Bruker APEXII CCD diffractometer
Absorption correction: multi-scan (*SADABS*; Sheldrick, 2004 ▶) *T*_min_ = 0.473, *T*_max_ = 0.521
5958 measured reflections
3683 independent reflections
2723 reflections with *I* > 2σ(*I*)
*R*_int_ = 0.026

#### Refinement

*R*[*F* ^2^ > 2σ(*F* ^2^)] = 0.038
*wR*(*F* ^2^) = 0.087
*S* = 1.05
3683 reflections
255 parameters
H-atom parameters constrained
Δρ_max_ = 0.39 e Å^−3^
Δρ_min_ = −0.46 e Å^−3^

### 

Data collection: *APEX2* (Bruker, 2004 ▶); cell refinement: *SAINT* (Bruker, 2004 ▶); data reduction: *SAINT*; program(s) used to solve structure: *SHELXS97* (Sheldrick, 2008 ▶); program(s) used to refine structure: *SHELXL97* (Sheldrick, 2008 ▶); molecular graphics: *SHELXTL* (Sheldrick, 2008 ▶); software used to prepare material for publication: *SHELXTL*.

## Supplementary Material

Crystal structure: contains datablock(s) global, I. DOI: 10.1107/S1600536811022513/hb5900sup1.cif

Structure factors: contains datablock(s) I. DOI: 10.1107/S1600536811022513/hb5900Isup2.hkl

Additional supplementary materials:  crystallographic information; 3D view; checkCIF report

## Figures and Tables

**Table 1:** Selected bond lengths (Å)

Cu1—N2	1.978 (3)
Cu1—N4	1.998 (3)
Cu1—N3	2.010 (3)
Cu1—N1	2.067 (3)
Cu1—Br1	2.5447 (7)

**Table 2:** Hydrogen-bond geometry (Å, °)

*D*—H⋯*A*	*D*—H	H⋯*A*	*D*⋯*A*	*D*—H⋯*A*
C2—H2⋯F1	0.93	2.49	3.413 (5)	173
C12—H12*B*⋯F3^i^	0.96	2.50	3.430 (5)	162
C15—H15⋯F2^ii^	0.93	2.40	3.252 (5)	152

Symmetry codes: (i) 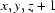; (ii) 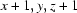.

**Fig. 1.**
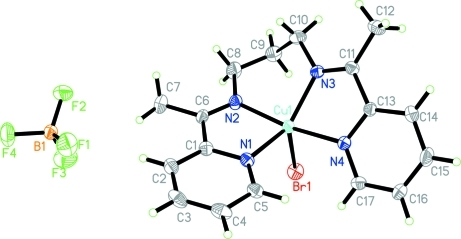
The molecular structure of (I) with 30% probability displacement ellipsoids.

## supplementary crystallographic information

### Comment

As a continuation of our work on the Schiff base copper(II) complexes (Liu, 2011), the title new copper complex, (I), is reported.

The title compound contains a mononuclear copper(II) complex cation and a fluoroborate anion, Fig. 1. The Cu^II^ atom in the complex is five-coordinated by the four N atoms of the Schiff base ligand, and by one bromide ion, forming a square-pyramidal geometry. The bond lengths (Table 1) related to the Cu atom are comparable with those observed in similar copper complexes with square-pyramidal geometry (Liu, 2011).

### Experimental

2-Acetylpyridine (0.2 mmol, 24.2 mg), propane-1,3-diamine (0.1 mmol, 7.4 mg), copper bromide (0.1 mmol, 22.3 mg), and ammonium fluoroborate (0.1 mmol, 10.5 mg) were mixed and stirred in methanol (20 ml) at reflux for 2 h, to give a blue solution. The solution was cooled to room temperature, and blue block-shaped single crystals of (I) were formed by slow evaporation of the solution in air.

### Refinement

H atoms were positioned geometrically (C–H = 0.93–0.97 Å) and refined using a riding model, with *U*_iso_(H) = 1.2*U*_eq_(C) and 1.5*U*_eq_(C_methyl_).

### Crystal data

**Table:**

[CuBr(C_17_H_20_N_4_)]BF_4_	*Z* = 2
*M**_r_* = 510.63	*F*(000) = 510
Triclinic, *P*1	*D*_x_ = 1.765 Mg m^−^^3^
*a* = 8.2508 (17) Å	Mo *K*α radiation, λ = 0.71073 Å
*b* = 8.9511 (18) Å	Cell parameters from 1747 reflections
*c* = 13.158 (3) Å	θ = 2.5–25.3°
α = 92.391 (2)°	µ = 3.26 mm^−^^1^
β = 94.847 (2)°	*T* = 298 K
γ = 96.422 (2)°	Block, blue
*V* = 960.9 (3) Å^3^	0.27 × 0.27 × 0.23 mm

### Data collection

**Table:**

Bruker APEXII CCD diffractometer	3683 independent reflections
Radiation source: fine-focus sealed tube	2723 reflections with *I* > 2σ(*I*)
graphite	*R*_int_ = 0.026
ω scans	θ_max_ = 26.0°, θ_min_ = 2.3°
Absorption correction: multi-scan (*SADABS*; Sheldrick, 2004)	*h* = −9→10
*T*_min_ = 0.473, *T*_max_ = 0.521	*k* = −11→10
5958 measured reflections	*l* = −16→11

### Refinement

**Table:**

Refinement on *F*^2^	Primary atom site location: structure-invariant direct methods
Least-squares matrix: full	Secondary atom site location: difference Fourier map
*R*[*F*^2^ > 2σ(*F*^2^)] = 0.038	Hydrogen site location: inferred from neighbouring sites
*wR*(*F*^2^) = 0.087	H-atom parameters constrained
*S* = 1.05	*w* = 1/[σ^2^(*F*_o_^2^) + (0.0353*P*)^2^ + 0.1409*P*] where *P* = (*F*_o_^2^ + 2*F*_c_^2^)/3
3683 reflections	(Δ/σ)_max_ = 0.001
255 parameters	Δρ_max_ = 0.39 e Å^−^^3^
0 restraints	Δρ_min_ = −0.46 e Å^−^^3^

### Special details

**Table:**

Geometry. All e.s.d.'s (except the e.s.d. in the dihedral angle between two l.s. planes) are estimated using the full covariance matrix. The cell e.s.d.'s are taken into account individually in the estimation of e.s.d.'s in distances, angles and torsion angles; correlations between e.s.d.'s in cell parameters are only used when they are defined by crystal symmetry. An approximate (isotropic) treatment of cell e.s.d.'s is used for estimating e.s.d.'s involving l.s. planes.
Refinement. Refinement of *F*^2^ against ALL reflections. The weighted *R*-factor *wR* and goodness of fit *S* are based on *F*^2^, conventional *R*-factors *R* are based on *F*, with *F* set to zero for negative *F*^2^. The threshold expression of *F*^2^ > σ(*F*^2^) is used only for calculating *R*-factors(gt) *etc*. and is not relevant to the choice of reflections for refinement. *R*-factors based on *F*^2^ are statistically about twice as large as those based on *F*, and *R*- factors based on ALL data will be even larger.

### Fractional atomic coordinates and isotropic or equivalent isotropic displacement parameters (Å^2^)

**Table:**

	*x*	*y*	*z*	*U*_iso_*/*U*_eq_	
Cu1	0.90467 (5)	0.78988 (5)	0.74936 (3)	0.03382 (14)	
Br1	1.14320 (5)	0.95979 (5)	0.69040 (3)	0.04830 (14)	
F1	0.5630 (4)	0.4076 (3)	0.2862 (2)	0.0787 (8)	
F2	0.4093 (3)	0.5940 (3)	0.2543 (2)	0.0754 (8)	
F3	0.6555 (3)	0.5941 (3)	0.1938 (2)	0.0870 (9)	
F4	0.4467 (4)	0.4290 (3)	0.1279 (2)	0.0792 (8)	
N1	0.9085 (3)	0.5870 (3)	0.6691 (2)	0.0318 (7)	
N2	0.7458 (4)	0.8146 (3)	0.6317 (2)	0.0370 (7)	
N3	0.7643 (3)	0.8783 (3)	0.8484 (2)	0.0339 (7)	
N4	1.0231 (3)	0.7455 (3)	0.8815 (2)	0.0343 (7)	
B1	0.5174 (6)	0.5077 (5)	0.2150 (4)	0.0410 (11)	
C1	0.8099 (4)	0.5758 (4)	0.5809 (3)	0.0333 (8)	
C2	0.7912 (5)	0.4481 (4)	0.5165 (3)	0.0410 (10)	
H2	0.7238	0.4432	0.4558	0.049*	
C3	0.8740 (5)	0.3273 (4)	0.5435 (3)	0.0490 (11)	
H3	0.8628	0.2403	0.5011	0.059*	
C4	0.9726 (5)	0.3373 (4)	0.6333 (3)	0.0463 (10)	
H4	1.0284	0.2570	0.6530	0.056*	
C5	0.9879 (5)	0.4694 (4)	0.6942 (3)	0.0388 (9)	
H5	1.0558	0.4764	0.7548	0.047*	
C6	0.7252 (4)	0.7110 (4)	0.5613 (3)	0.0338 (8)	
C7	0.6216 (5)	0.7181 (5)	0.4626 (3)	0.0549 (12)	
H7A	0.5098	0.7227	0.4763	0.082*	
H7B	0.6287	0.6299	0.4198	0.082*	
H7C	0.6602	0.8062	0.4285	0.082*	
C8	0.6550 (5)	0.9469 (4)	0.6228 (3)	0.0506 (11)	
H8A	0.5399	0.9130	0.6050	0.061*	
H8B	0.6943	1.0060	0.5678	0.061*	
C9	0.6728 (5)	1.0446 (4)	0.7192 (3)	0.0496 (11)	
H9A	0.6033	1.1245	0.7103	0.060*	
H9B	0.7852	1.0911	0.7308	0.060*	
C10	0.6290 (5)	0.9614 (5)	0.8124 (3)	0.0478 (11)	
H10A	0.6076	1.0324	0.8660	0.057*	
H10B	0.5305	0.8919	0.7956	0.057*	
C11	0.7903 (4)	0.8446 (4)	0.9409 (3)	0.0339 (8)	
C12	0.6860 (5)	0.8754 (5)	1.0249 (3)	0.0546 (12)	
H12A	0.7435	0.9522	1.0718	0.082*	
H12B	0.6614	0.7850	1.0601	0.082*	
H12C	0.5860	0.9089	0.9966	0.082*	
C13	0.9414 (4)	0.7715 (4)	0.9639 (3)	0.0316 (8)	
C14	0.9996 (5)	0.7370 (5)	1.0603 (3)	0.0447 (10)	
H14	0.9410	0.7538	1.1163	0.054*	
C15	1.1478 (5)	0.6765 (5)	1.0722 (3)	0.0492 (11)	
H15	1.1893	0.6518	1.1364	0.059*	
C16	1.2322 (5)	0.6535 (4)	0.9889 (3)	0.0434 (10)	
H16	1.3313	0.6130	0.9960	0.052*	
C17	1.1684 (4)	0.6912 (4)	0.8944 (3)	0.0403 (9)	
H17	1.2275	0.6788	0.8381	0.048*	

### Atomic displacement parameters (Å^2^)

**Table:**

	*U*^11^	*U*^22^	*U*^33^	*U*^12^	*U*^13^	*U*^23^
Cu1	0.0384 (3)	0.0369 (3)	0.0276 (3)	0.0138 (2)	−0.00162 (19)	0.00188 (19)
Br1	0.0507 (3)	0.0410 (3)	0.0523 (3)	−0.00041 (19)	0.0050 (2)	0.00788 (19)
F1	0.103 (2)	0.0687 (19)	0.0661 (19)	0.0260 (16)	−0.0107 (16)	0.0165 (15)
F2	0.0599 (16)	0.101 (2)	0.0701 (18)	0.0396 (16)	0.0013 (14)	−0.0089 (16)
F3	0.0684 (19)	0.080 (2)	0.112 (3)	−0.0095 (16)	0.0233 (17)	0.0126 (18)
F4	0.096 (2)	0.0772 (19)	0.0576 (18)	0.0063 (16)	−0.0192 (15)	−0.0198 (15)
N1	0.0362 (17)	0.0312 (17)	0.0286 (17)	0.0035 (13)	0.0064 (13)	0.0045 (13)
N2	0.0417 (19)	0.0374 (19)	0.0320 (18)	0.0093 (15)	−0.0039 (14)	0.0064 (15)
N3	0.0322 (17)	0.0351 (17)	0.0354 (18)	0.0110 (13)	0.0012 (14)	−0.0001 (14)
N4	0.0341 (17)	0.0398 (18)	0.0299 (17)	0.0095 (14)	0.0001 (14)	0.0019 (14)
B1	0.041 (3)	0.043 (3)	0.038 (3)	0.010 (2)	−0.003 (2)	−0.003 (2)
C1	0.033 (2)	0.035 (2)	0.031 (2)	−0.0037 (16)	0.0076 (16)	0.0049 (17)
C2	0.039 (2)	0.043 (2)	0.039 (2)	−0.0075 (18)	0.0090 (18)	−0.0057 (19)
C3	0.058 (3)	0.034 (2)	0.055 (3)	−0.009 (2)	0.026 (2)	−0.011 (2)
C4	0.056 (3)	0.030 (2)	0.058 (3)	0.0101 (19)	0.024 (2)	0.008 (2)
C5	0.046 (2)	0.038 (2)	0.035 (2)	0.0116 (18)	0.0108 (18)	0.0052 (18)
C6	0.0279 (19)	0.045 (2)	0.028 (2)	−0.0011 (16)	0.0007 (15)	0.0060 (18)
C7	0.054 (3)	0.065 (3)	0.042 (3)	0.004 (2)	−0.014 (2)	0.001 (2)
C8	0.061 (3)	0.045 (3)	0.047 (3)	0.020 (2)	−0.008 (2)	0.009 (2)
C9	0.058 (3)	0.040 (2)	0.054 (3)	0.025 (2)	−0.002 (2)	0.008 (2)
C10	0.042 (2)	0.049 (3)	0.056 (3)	0.024 (2)	0.003 (2)	0.003 (2)
C11	0.036 (2)	0.033 (2)	0.034 (2)	0.0071 (16)	0.0058 (17)	0.0003 (17)
C12	0.053 (3)	0.071 (3)	0.045 (3)	0.023 (2)	0.015 (2)	0.003 (2)
C13	0.0322 (19)	0.032 (2)	0.030 (2)	0.0042 (16)	−0.0006 (16)	−0.0007 (16)
C14	0.048 (2)	0.058 (3)	0.030 (2)	0.010 (2)	0.0038 (18)	0.0015 (19)
C15	0.056 (3)	0.058 (3)	0.034 (2)	0.014 (2)	−0.010 (2)	0.010 (2)
C16	0.039 (2)	0.052 (3)	0.040 (2)	0.0129 (19)	−0.0053 (19)	0.0042 (19)
C17	0.037 (2)	0.047 (2)	0.039 (2)	0.0144 (18)	0.0048 (18)	0.0019 (18)

### Geometric parameters (Å, °)

**Table:**

Cu1—N2	1.978 (3)	C6—C7	1.501 (5)
Cu1—N4	1.998 (3)	C7—H7A	0.9600
Cu1—N3	2.010 (3)	C7—H7B	0.9600
Cu1—N1	2.067 (3)	C7—H7C	0.9600
Cu1—Br1	2.5447 (7)	C8—C9	1.498 (6)
F1—B1	1.381 (5)	C8—H8A	0.9700
F2—B1	1.363 (5)	C8—H8B	0.9700
F3—B1	1.360 (5)	C9—C10	1.511 (5)
F4—B1	1.370 (5)	C9—H9A	0.9700
N1—C5	1.339 (4)	C9—H9B	0.9700
N1—C1	1.354 (4)	C10—H10A	0.9700
N2—C6	1.270 (4)	C10—H10B	0.9700
N2—C8	1.474 (4)	C11—C13	1.486 (5)
N3—C11	1.272 (4)	C11—C12	1.492 (5)
N3—C10	1.467 (4)	C12—H12A	0.9600
N4—C17	1.343 (4)	C12—H12B	0.9600
N4—C13	1.349 (4)	C12—H12C	0.9600
C1—C2	1.381 (5)	C13—C14	1.378 (5)
C1—C6	1.485 (5)	C14—C15	1.392 (5)
C2—C3	1.385 (5)	C14—H14	0.9300
C2—H2	0.9300	C15—C16	1.367 (5)
C3—C4	1.371 (6)	C15—H15	0.9300
C3—H3	0.9300	C16—C17	1.380 (5)
C4—C5	1.388 (5)	C16—H16	0.9300
C4—H4	0.9300	C17—H17	0.9300
C5—H5	0.9300		
			
N2—Cu1—N4	167.79 (12)	C6—C7—H7B	109.5
N2—Cu1—N3	92.15 (12)	H7A—C7—H7B	109.5
N4—Cu1—N3	79.82 (12)	C6—C7—H7C	109.5
N2—Cu1—N1	80.08 (12)	H7A—C7—H7C	109.5
N4—Cu1—N1	99.56 (12)	H7B—C7—H7C	109.5
N3—Cu1—N1	137.46 (11)	N2—C8—C9	112.8 (3)
N2—Cu1—Br1	96.74 (9)	N2—C8—H8A	109.0
N4—Cu1—Br1	95.17 (9)	C9—C8—H8A	109.0
N3—Cu1—Br1	118.16 (9)	N2—C8—H8B	109.0
N1—Cu1—Br1	104.32 (8)	C9—C8—H8B	109.0
C5—N1—C1	118.4 (3)	H8A—C8—H8B	107.8
C5—N1—Cu1	129.4 (2)	C8—C9—C10	113.9 (4)
C1—N1—Cu1	112.2 (2)	C8—C9—H9A	108.8
C6—N2—C8	120.0 (3)	C10—C9—H9A	108.8
C6—N2—Cu1	117.1 (2)	C8—C9—H9B	108.8
C8—N2—Cu1	122.9 (3)	C10—C9—H9B	108.8
C11—N3—C10	123.0 (3)	H9A—C9—H9B	107.7
C11—N3—Cu1	115.8 (2)	N3—C10—C9	109.7 (3)
C10—N3—Cu1	120.8 (2)	N3—C10—H10A	109.7
C17—N4—C13	119.2 (3)	C9—C10—H10A	109.7
C17—N4—Cu1	126.9 (2)	N3—C10—H10B	109.7
C13—N4—Cu1	113.8 (2)	C9—C10—H10B	109.7
F3—B1—F2	110.9 (4)	H10A—C10—H10B	108.2
F3—B1—F4	109.5 (4)	N3—C11—C13	114.8 (3)
F2—B1—F4	110.2 (4)	N3—C11—C12	125.8 (3)
F3—B1—F1	107.5 (3)	C13—C11—C12	119.4 (3)
F2—B1—F1	109.3 (4)	C11—C12—H12A	109.5
F4—B1—F1	109.3 (4)	C11—C12—H12B	109.5
N1—C1—C2	121.8 (3)	H12A—C12—H12B	109.5
N1—C1—C6	114.1 (3)	C11—C12—H12C	109.5
C2—C1—C6	124.1 (3)	H12A—C12—H12C	109.5
C1—C2—C3	119.1 (4)	H12B—C12—H12C	109.5
C1—C2—H2	120.4	N4—C13—C14	121.6 (3)
C3—C2—H2	120.4	N4—C13—C11	114.1 (3)
C4—C3—C2	119.3 (4)	C14—C13—C11	124.3 (3)
C4—C3—H3	120.4	C13—C14—C15	118.5 (4)
C2—C3—H3	120.4	C13—C14—H14	120.7
C3—C4—C5	118.9 (4)	C15—C14—H14	120.7
C3—C4—H4	120.6	C16—C15—C14	119.7 (4)
C5—C4—H4	120.6	C16—C15—H15	120.1
N1—C5—C4	122.5 (4)	C14—C15—H15	120.1
N1—C5—H5	118.8	C15—C16—C17	119.1 (4)
C4—C5—H5	118.8	C15—C16—H16	120.4
N2—C6—C1	116.3 (3)	C17—C16—H16	120.4
N2—C6—C7	124.1 (4)	N4—C17—C16	121.7 (3)
C1—C6—C7	119.6 (3)	N4—C17—H17	119.2
C6—C7—H7A	109.5	C16—C17—H17	119.2

### Hydrogen-bond geometry (Å, °)

**Table:**

*D*—H···*A*	*D*—H	H···*A*	*D*···*A*	*D*—H···*A*
C2—H2···F1	0.93	2.49	3.413 (5)	173
C12—H12B···F3^i^	0.96	2.50	3.430 (5)	162
C15—H15···F2^ii^	0.93	2.40	3.252 (5)	152

Symmetry codes: (i) *x*, *y*, *z*+1; (ii) *x*+1, *y*, *z*+1.

## References

[bb1] Bruker (2004). *APEX2* and *SAINT* Bruker AXS Inc., Madison, Wisconsin, USA.

[bb2] Liu, L.-J. (2011). *Acta Cryst.* E**67**, m876–m877. 10.1107/S1600536811021040 PMC3152035 21836871

[bb3] Sheldrick, G. M. (2004). *SADABS* University of Göttingen, Germany.

[bb4] Sheldrick, G. M. (2008). *Acta Cryst.* A**64**, 112–122. 10.1107/S0108767307043930 18156677

